# Genome-wide analysis of RNA-chromatin interactions in lizards as a mean for functional lncRNA identification

**DOI:** 10.1186/s12864-023-09545-5

**Published:** 2023-08-07

**Authors:** Mariela Tenorio, Joanna Serwatowska, Selene L. Fernandez-Valverde, Katarzyna Oktaba, Diego Cortez

**Affiliations:** 1 Center for Genome Sciences, National Autonomous University of Mexico (UNAM), Cuernavaca, Mexico; 2 Center for Research and Advanced Studies (Cinvestav), Irapuato, Mexico; 3 Present Address: School of Biotechnology and Biomolecular Sciences and the RNA Institute, The University of New South Wales, Sydney, NSW 2052 Australia

**Keywords:** Chromatin, Long non-coding RNA, *Anolis carolinensis*, Chromatin-associated RNA sequencing

## Abstract

**Background:**

Long non-coding RNAs (lncRNAs) are defined as transcribed molecules longer than 200 nucleotides with little to no protein-coding potential. LncRNAs can regulate gene expression of nearby genes (*cis*-acting) or genes located on other chromosomes (*trans*-acting). Several methodologies have been developed to capture lncRNAs associated with chromatin at a genome-wide level. Analysis of RNA-DNA contacts can be combined with epigenetic and RNA-seq data to define potential lncRNAs involved in the regulation of gene expression.

**Results:**

We performed Chromatin Associated RNA sequencing (ChAR-seq) in *Anolis carolinensis* to obtain the genome-wide map of the associations that RNA molecules have with chromatin. We analyzed the frequency of DNA contacts for different classes of RNAs and were able to define *cis*- and *trans*-acting lncRNAs. We integrated the ChAR-seq map of RNA-DNA contacts with epigenetic data for the acetylation of lysine 16 on histone H4 (H4K16ac), a mark connected to actively transcribed chromatin in lizards. We successfully identified three *trans*-acting lncRNAs significantly associated with the H4K16ac signal, which are likely involved in the regulation of gene expression in *A. carolinensis*.

**Conclusions:**

We show that the ChAR-seq method is a powerful tool to explore the RNA-DNA map of interactions. Moreover, in combination with epigenetic data, ChAR-seq can be applied in non-model species to establish potential roles for predicted lncRNAs that lack functional annotations.

**Supplementary Information:**

The online version contains supplementary material available at 10.1186/s12864-023-09545-5.

## Background

The eukaryotic cell is home to a plethora of non-coding RNAs, among which ribosomal RNAs, small nuclear RNAs, and small nucleolar RNAs are the most abundant. Ribosomal RNAs play a crucial role in translating messenger RNAs, while small nuclear RNAs are essential for gene splicing, and small nucleolar RNAs guide chemical modifications of other RNA molecules. The most enigmatic group of non-coding RNAs is long-non coding RNAs (lncRNAs) [1], encompassing RNA molecules longer than 200 nucleotides that lack protein-coding potential. Transcriptomic studies have identified thousands of lncRNAs that may possess functional roles in humans and mice [2–10]. Large-scale screenings have associated many of these lncRNAs with regulatory functions [11].

LncRNAs can be categorized into two groups based on their regulatory activity. *Cis*-acting lncRNAs regulate gene expression on the same chromosome from which they are transcribed, whereas *trans*-acting lncRNAs regulate gene transcription on different chromosomes. Some extensively studied *cis*-acting lncRNAs in vertebrates include *XIST* [12–14], *RSX* [15], and *ROX2* [16], which regulate the expression levels of entire X chromosomes in placental mammals, marsupials, and the fruit fly, respectively. Other characterized lncRNAs can regulate genetic imprinting [17], recruit protein complexes that modify chromatin [18], or influence the expression of remote genes [19]. Although a few *trans*-acting lncRNAs have been experimentally studied, their number remains limited. Notably, *HOTAIR* [20, 21], a lncRNA known to silence the *HOXD* gene by recruiting the Polycomb Repressive Complex 2 has faced challenges regarding its *trans*-activity following a recent study that analyzed *HOTAIR* knockout mice [22]. Another example is FIRRE, a trans-acting lncRNA involved in hematopoiesis [23].

For more than two decades, hybridization capture methods have been the standard technique for identifying the DNA and proteins associated with specific lncRNA [24]. These methods, known as one-to-all approaches, employ biotinylated DNA probes to selectively purify a lncRNA that has been cross-linked to their adjacent DNA and binding proteins. The most renowned techniques include Chromatin Isolation by RNA Purification (ChIRP) [25], Capture Hybridization Analysis of RNA Targets (CHART) [26], and RNA antisense purification (RAP) [27].

Recently, four all-to-all approaches have emerged to capture all possible interactions between RNA molecules and chromatin. These methodologies are designed to provide comprehensive insights into RNA-genome interactions. The four methods are MARGI (Mapping RNA–Genome Interactions) [28], ChAR-seq (Chromatin-Associated RNA sequencing) [29, 30], GRID-seq (Global RNA Interaction with DNA sequencing) [31], and RADICL-seq (RNA And DNA Interacting Complexes Ligated and sequenced) [32]. These methodologies involve capturing RNAs in contact with DNA by employing specific short linkers that ligate an RNA fragment to an adjacent DNA fragment. Both MARGI and CHAR-seq enable the sequencing of long RNA-DNA tags. In a successful application of MARGI, researchers used human cells to demonstrate that *XIST* exhibits long-range binding sites along the female X chromosome [28]. Similarly, ChAR-seq was employed in *Drosophila* to unveil the detailed map of RNA-DNA contacts of *ROX2* along the X chromosome of males [30].

While these all-to-all techniques have proven successful in model species such as humans, mice, and *Drosophila*, RNA-DNA contact maps have yet to be explored in other species. In recent years, the number of reptile genomes deposited in public databases has increased by over 600% (from 17 genomes before 2018 to 123 genomes between 2018 and 2023). However, gene annotations in these genomes typically rely on automated modeling of gene predictions based on protein-coding genes from species with curated annotations. In some cases, such as the reference genome of the green anole lizard, *Anolis carolinensis*, transcriptomic data was utilized to enhance the annotations of coding and non-coding genes [33]. The current version of the genome of *A. carolinensis* contains 3,176 lncRNAs (https://www.ensembl.org/Anolis_carolinensis/Info/Annotation), yet most of them lack functional information. In this study, we hypothesized that ChAR-seq-like methods could aid in identifying potential regulatory lncRNAs in genomes where they have been predicted. It should be noted that the ChAR-seq method can provide information about RNA molecules that interact with DNA but is unable to report interactions with other molecules, such as proteins. Therefore, we applied the ChAR-seq method in *A. carolinensis* to investigate the overall map of interactions between RNA molecules and chromatin. We characterized the frequencies of contact for different classes of RNAs and annotate *cis*- and *trans*-acting lncRNAs. By correlating the ChAR-seq results with ChIP-seq data for the acetylation of lysine 16 on histone H4 (H4K16ac) epigenetic mark, we identified three lncRNAs with *trans*-activity that likely play a role in gene expression regulation in *A. carolinensis*.

## Results

### Variations in the frequency of associations between RNA and chromatin

To investigate the interactions between RNA molecules and chromatin in *A. carolinensis*, we employed the ChAR-seq method on two adult liver samples. By utilizing a specialized short linker, we captured RNA molecules in contact with chromatin and sequenced a total of 1,020,074,230 and 9,96,050,284 reads from the two biological replicates. The paired reads were then mapped to generate a comprehensive genome-wide map of RNA-chromatin interactions.

**Fig. 1:**
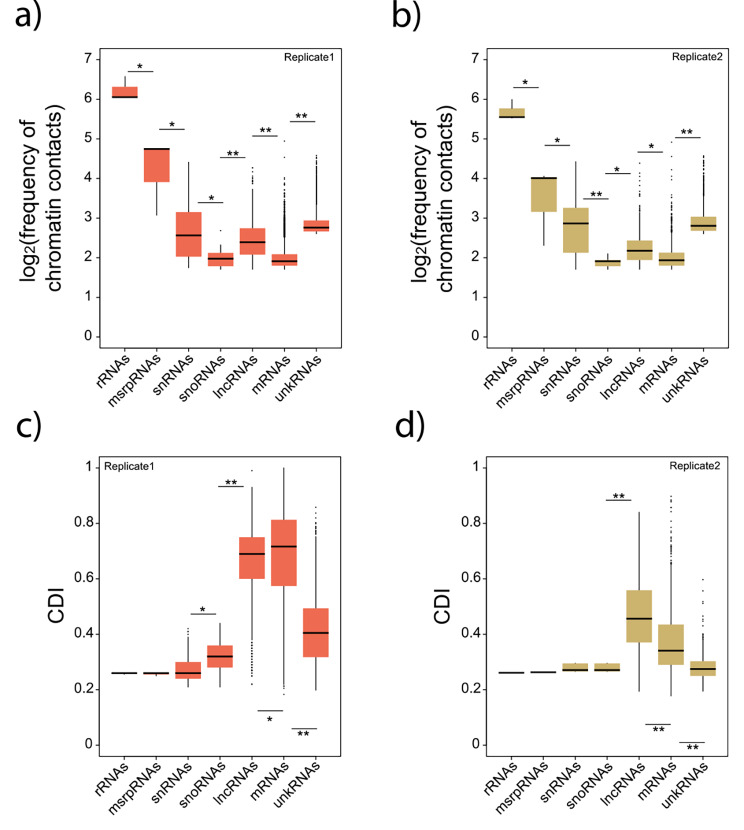
**Chromatin contacts for seven different classes of RNAs**. (**a**) Boxplots representing the log_2_-transformed ratio of the frequency of chromatin contacts for seven different types of RNA molecules. (**b**) Same as (a) for replicate 2. (**c**) Boxplot representing the values of the Contact Distribution Index (CDI) for seven different classes of RNA molecules. (**d**) Same as (c) for replicate 2. N values for replicate 1 are ribosomal RNAs, 3; metazoan signal recognition particle RNAs, 3; small RNAs, 70; small nucleolar RNAs, 33; long non-coding RNAs, 1188, messenger RNAs, 3724, unannotated RNAs, 3338. N values for replicate 2 are ribosomal RNAs, 3; metazoan signal recognition particle RNAs, 3; small RNAs, 6; small nucleolar RNAs, 5; long non-coding RNAs, 756, messenger RNAs, 769, unannotated RNAs,756. Data for lncRNAs, mRNAs, and unkRNAs are limited to chromosomes 1–6. Error bars, maximum and minimum values, excluding outliers. Significant differences, Mann-Whitney U test; * represents *P* < 0.01, ** represents *P* < 0.001. *P*-values were corrected using the Benjamini-Hochberg method

We analyzed unique interactions for each class of RNA present in the cell. As expected, highly abundant RNA molecules, such as ribosomal RNAs (rRNAs), were overrepresented in our results. Based on the gene annotations available for *A. carolinenesis*, we found that ribosomal RNAs (rRNAs) accounted for 70% of the interactions, whereas long non-coding RNAs (lncRNAs) represented 14%, messenger RNAs (mRNAs) 13%, small nuclear RNAs (snRNAs) 2%, the metazoan signal recognition particle RNA (metazoan srpRNA) 1%, and small nucleolar RNAs (snoRNAs) 0.04% (Fig. 1a,b). The rRNA genes exhibited interactions with numerous chromatin sites (average contacts per gene = 1,132,775), whereas the three annotated metazoan srpRNAs showed an average of 55,000 contacts with chromatin across the genome. The frequency of contacts for other RNA types ranged between 50 and 80,000 (Fig. 1a,b), with consistent patterns across the two replicates (Fig. 1a,b). Given the potential presence of unannotated non-coding genes in the *A. carolinensis* genome, we analyzed our RNA-chromatin interactions dataset using a sliding window of 10Kb. This analysis revealed 9,000 transcribed regions that do not overlap with known coding or non-coding genes, exhibiting between 500 and 87,000 chromatin interactions (Fig. 1a,b; unkRNA).

Subsequently, we determined whether the RNA-chromatin contacts were localized within the same chromosomes (intra-chromosomal) or involved contacts across different chromosomes (inter-chromosomal). To assess this, we introduced a Contact Distribution Index (CDI), which was calculated by dividing the number of contacts on the chromosome with the highest interaction count by the total number of contacts across all chromosomes; CDI values around 0.2–0.3 indicated interactions spread across many chromosomes, while CDI > 0.4 indicated a bias toward fewer chromosomes. To ensure the reliability of the interactions in *cis* and *trans*, we focused on genes located on the assembled macro-chromosomes (1 to 6) and discarded the short fragments (scaffolds) in the *A. carolinensis* reference genome.

**Fig. 2:**
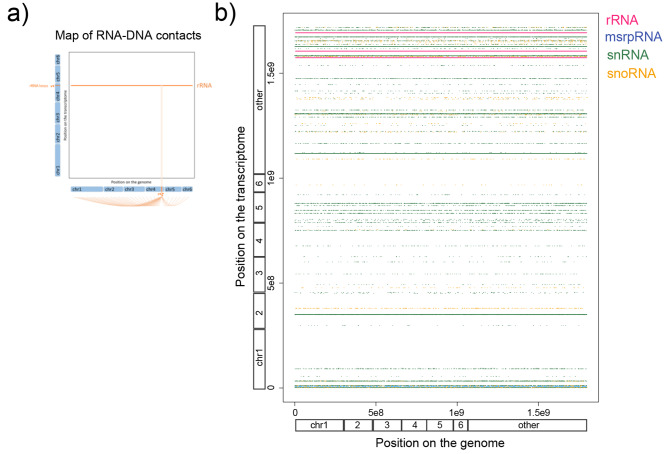
**Contact map of abundant non-coding RNAs between the transcriptome and the genome**. (**a**) Illustration to help explain the horizontal lines in panel b; these lines represent the contacts between a single locus on the transcriptome with multiple loci on the genome. (**b**) Dot-plot representation of specific non-coding RNAs loci on the transcriptome (Y-axis) and their multiple genomic contacts (X-axis). The positions on the genome and transcriptome correspond to the concatenated chromosomes 1 to 6 (indicated), followed by the linkage groups and the unassembled scaffolds ordered alphabetically (indicated as other). Ribosomal RNAs are in pink, the metazoan signal recognition particle RNAs in blue, small RNAs in green, and small nucleolar RNAs in orange

Our analysis revealed that rRNAs, metazoan srpRNAs, snRNAs, and snoRNAs displayed lower CDI values (Fig. 1c,d), indicating widespread contacts across the entire genome (Fig. 2a,b). This finding aligns with expectations, considering that ribosomal RNAs are the most abundant type of RNA molecules within eukaryotic cell nucleoli. Similarly, snRNAs and snoRNAs, involved in mRNA splicing or RNA chemical modifications, respectively, interact with numerous genomic regions. In contrast, lncRNAs and mRNAs exhibited higher CDI values (Fig. 1c,d), suggesting a predominance of intra-chromosomal contacts. Notably, unkRNAs showed lower CDI values (Fig. 1c,d), implying the absence of lncRNAs or mRNAs within these transcribed regions. In total, our analysis identified 2,282 lncRNAs in replicates 1 and 2 combined, representing 71.8% of the 3,176 lncRNAs annotated in the *A. carolinensis* genome.

### C *is*-acting and *trans*-acting lncRNAs

**Fig. 3:**
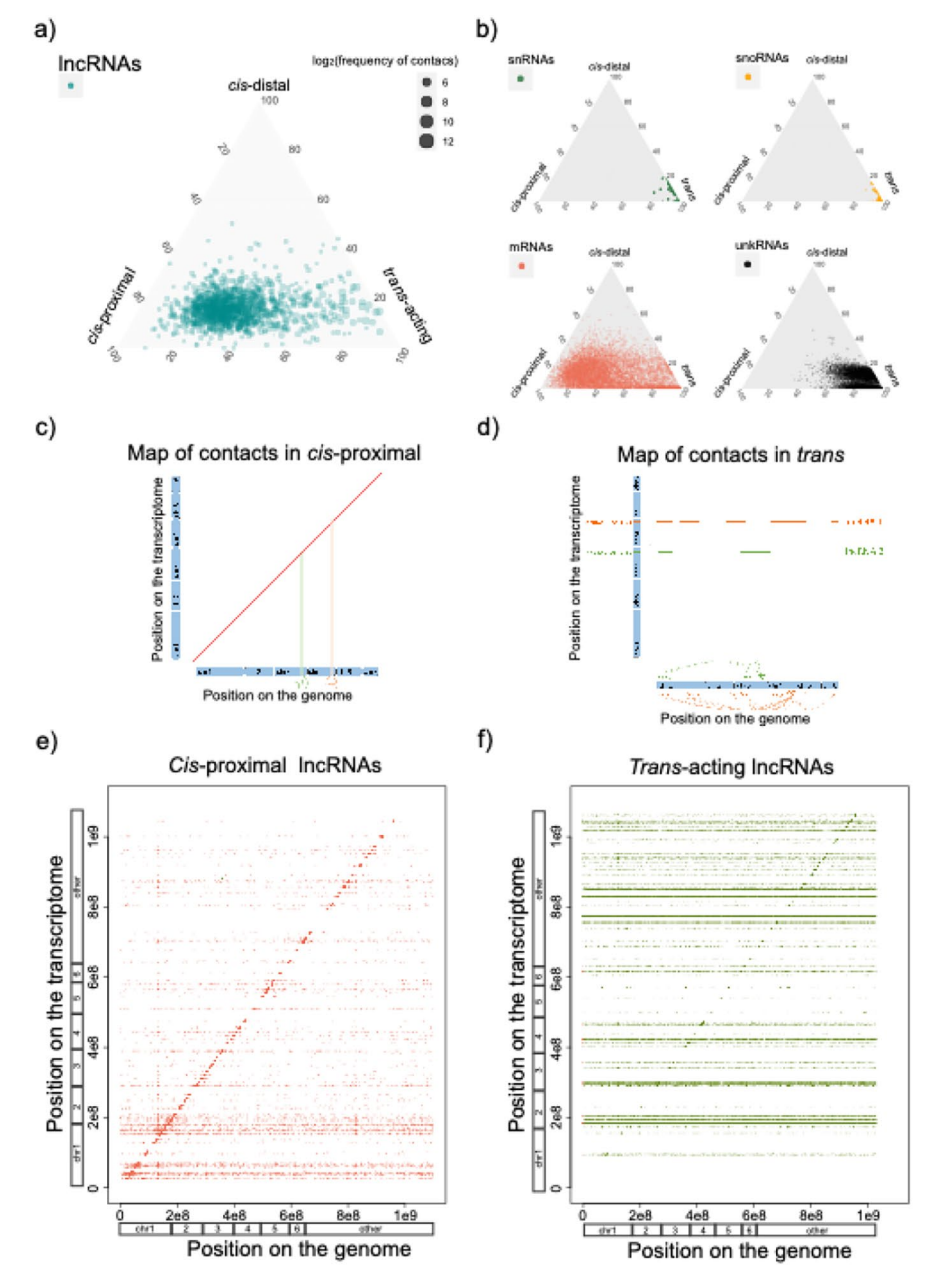
**Contact map of*cis*-acting and*trans*-acting lncRNAs between the transcriptome and the genome**. (**a**) Ternary plots representing the type of contacts of lncRNAs; *cis*-proximal (< 10 Kb around the gene loci), *cis*-distal (> 10 Kb on the same chromosome), *trans*-acting (in other chromosomes). Dot sizes are defined by log2 of the frequency of contacts. (**b**) Same as (a) for small RNAs, small nucleolar RNAs, messenger RNAs, and unannotated RNAs; rRNAs and msrpRNAs are not shown since their contacts are > 99.9% *trans*-acting. (**c**) Illustration to help explain the map of RNA-DNA contacts in *cis*-proximal. (**d**) Illustration to help explain the map of RNA-DNA contacts in *trans* (**e**) *Cis*-proximal lncRNAs have most of their RNA-DNA contacts within their loci, which explains the diagonal line on the dot plot. The dot-plot represents specific lncRNAs loci on the transcriptome (Y-axis) and their multiple genomic contacts (X-axis). (**f**) *Trans*-acting lncRNAs have most of their contacts on other chromosomes, which explains the horizontal lines on the dot plot. Same as (c) for *trans*-acting lncRNAs. The positions on the genome and transcriptome correspond to the concatenated chromosomes 1 to 6 (indicated), followed by the linkage groups and the unassembled scaffolds ordered alphabetically (indicated as other)

ChAR-seq data provides valuable insights into distinguishing between lncRNAs that interact with loci in close proximity (*cis*-proximal) or distantly (*cis*-distal) on the same chromosome from which they are transcribed, as well as lncRNAs that have interactions with other chromosomes (*trans*-acting). To examine these types of interactions, we analyzed the lncRNAs on chromosomes 1 to 6 and estimated the percentage of *cis*-proximal, *cis*-distant, and *trans*-acting contacts. Notably, lncRNAs exhibited a gradient distribution between *cis*-proximal and *trans*-acting interactions, with a substantial bias towards *cis*-proximal interactions (Fig. 3a). This distinctive sets lncRNAs apart from other classes of RNA molecules (Fig. 3b). Specifically, approximately, 87.5% (n = 1040) of lncRNAs displayed over 50% of their contacts in the *cis*-proximal category (Fig. 3c,e; Supplementary Table 1), while 12.5% (n = 148) exhibited over 50% of their interactions in the *trans*-acting category (Fig. 3d,f; Supplementary Table 1).

**Fig. 4:**
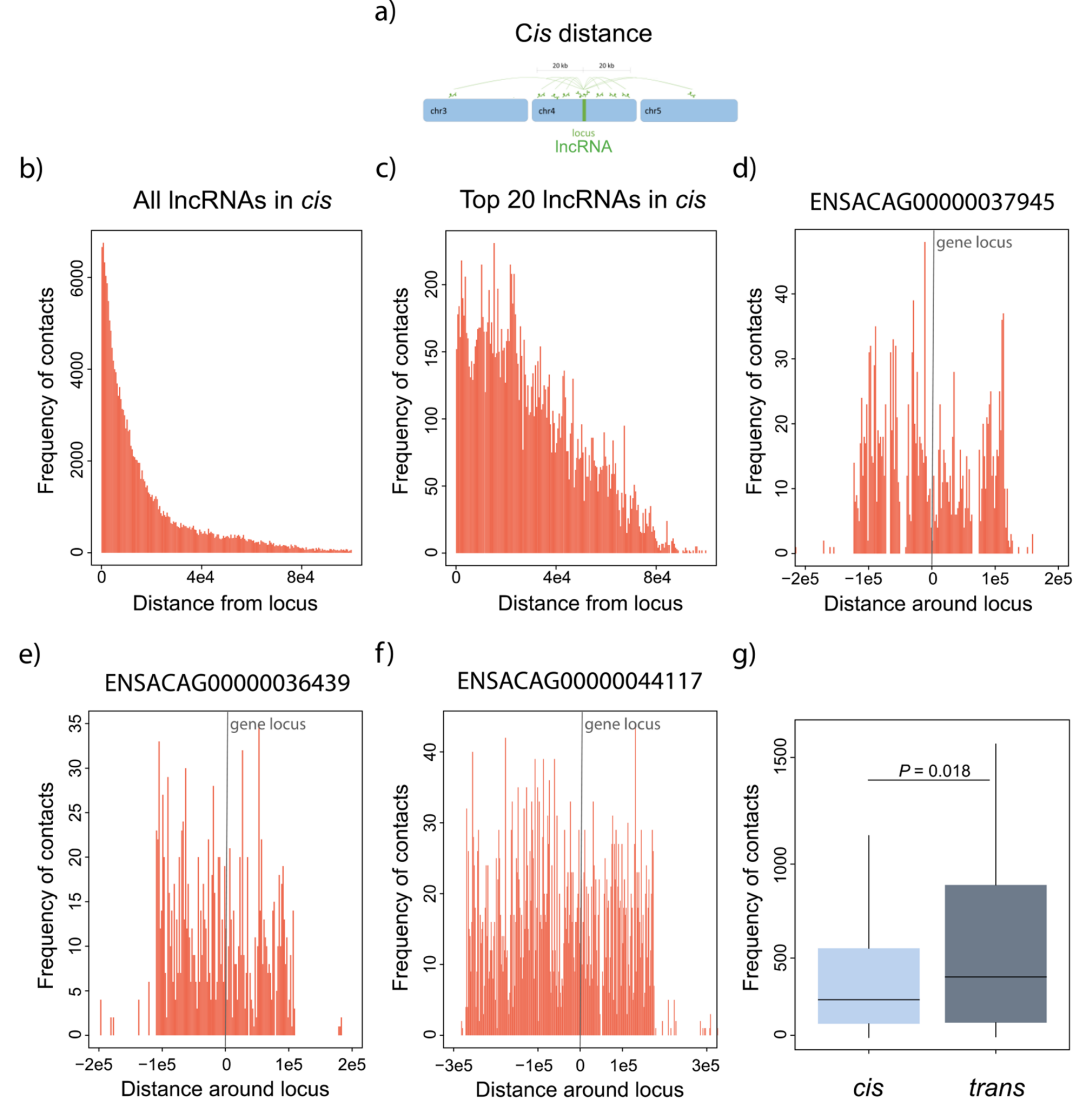
**Range and frequency of RNA-DNA contacts for*cis*-acting lncRNAs**. (**a**) Illustration to help explain the RNA-DNA contacts in *cis*. (**b**) Frequency and range of the RNA-DNA contacts (histograms in orange) for all *cis*-acting lncRNAs. (**c**) Same as in (b) but for the top twenty lncRNAs. (**d-f**) Three examples of lncRNAs and their frequency and range of contacts (histograms in orange) around their loci. (**g**) Frequency of contacts between *cis*-proximal and *trans*-acting lncRNAs. Significant differences, Mann-Whitney U test. Error bars, maximum and minimum values, excluding outliers. N values: *cis*-proximal, 1040; *trans*-acting 148

For 98% of the *cis*-acting lncRNAs, the majority of their chromatin contacts clustered within 20 Kb around the transcription locus of the lncRNA (Fig. 4a,b), aligning with the gene body of the lncRNA. However, upon narrowing our analysis to the top twenty *cis*-acting lncRNAs with the highest number of contacts, the range of interactions increased to approximately 40 Kb around the lncRNA locus (Fig. 4c), extending beyond the boundaries of the gene body. Notably, only when examining the top five *cis*-acting lncRNAs with the most contacts, the range of interactions further increased to 100 to 300 Kb around the lncRNA locus (Fig. 4d-f), encompassing nearby genes. Overall, our data revealed a substantial number of lncRNAs with contacts at their transcription sites, likely representing nascent transcripts.

**Fig. 5:**
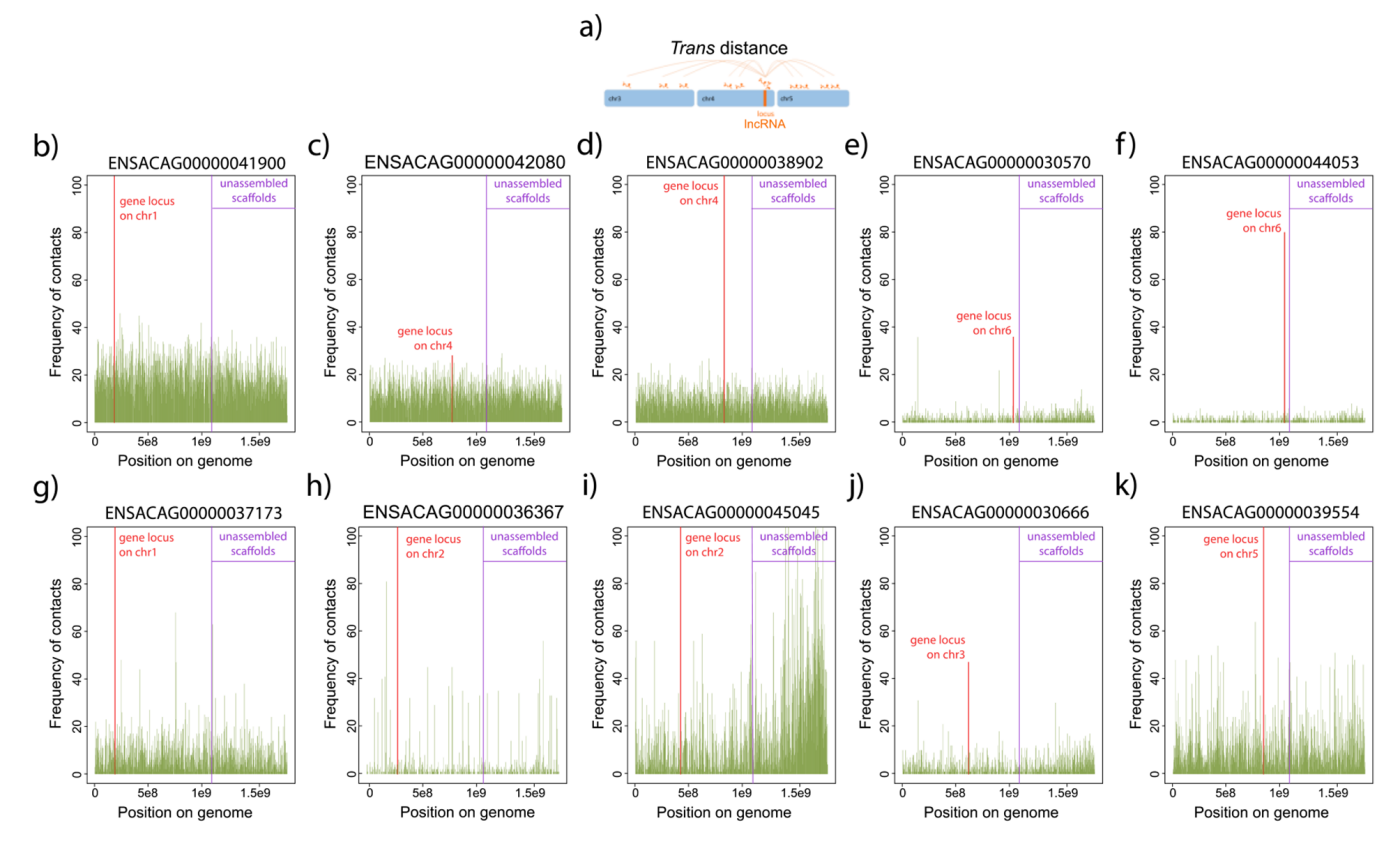
**Frequency of RNA-DNA contacts for*trans*-acting lncRNAs.** (**a**) Illustration to help explain the RNA-DNA contacts in *trans*. (**b-k**) Frequency of contacts (histograms in green) across the genome for the top ten lncRNAs with the largest number of contacts in *trans*. The positions on the genome (X-axis) correspond to the concatenated chromosomes 1 to 6, followed by the linkage groups and unassembled scaffolds ordered alphabetically. Purple lines indicate the boundary between the assembled and unassembled parts of the genome. The frequency of contacts at the lncRNAs locus is indicated by the orange histograms; the chromosome where each lncRNA is found is also indicated

Furthermore, we observed that *trans*-acting lncRNAs exhibited a larger number of chromatin interactions compared to *cis*-acting lncRNAs (Fig. 4g). Focusing on the top ten *trans*-acting lncRNAs, we discovered that they have interactions with all chromosomes, in addition to displaying a peak of interactions at their locus (Fig. 5a-k). Some of these *trans*-acting lncRNAs showed interactions throughout the genome, while others displayed interactions with specific genomic regions (Fig. 5g-k). Notably, examples such as ENSACAG00000045045 (Fig. 5i) and ENSACAG00000030666 (Fig. 5j) exhibited an enrichment of chromatin contacts at unassembled scaffolds. Conversely, ENSACAG00000036367 (Fig. 5h) and ENSACAG00000039554 (Fig. 5k) displayed discrete peaks of interactions distributed along the genome.

### Three *trans*-acting lncRNAs exhibit significant associations with the H4K16 acetylation signal

To investigate the potential role of trans-acting lncRNAs in gene expression regulation, we examined the top *trans*-acting lncRNAs and compared their chromatin interaction profiles against ChIP-seq data for the H4K16ac. In the green anole, H4K16ac is known to be enriched at transcription start sites and associated with active transcription [34]. We employed ChIP-seq data generated for both liver (the same tissue used for ChAR-seq) and brain [34]. We assessed whether the RNA-DNA contact regions of lncRNAs displayed a higher coverage of the H4K16ac mark compared to a randomized set of RNA-DNA contacts. Conversely, if a lncRNA is not associated with the H4K16ac signal, the enrichment for H4K16ac will not differ significantly from a randomized set of RNA-DNA contacts.

**Fig. 6:**
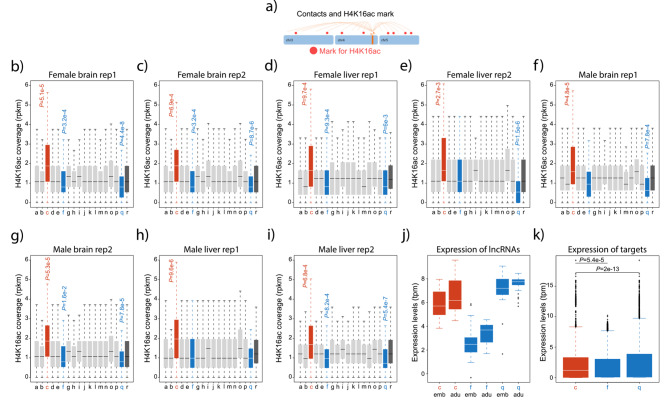
**H4K16ac coverage at chromatin contacts for the top*trans*-acting lncRNAs**. (**a**) Illustration to help explain the RNA-DNA contacts and their association with the H4K16ac mark. (**b-e**) Normalized coverage of the H4K16ac mark (RPKM) in female and male livers at the positions where lncRNAs interact with chromatin. The identity of the *trans*-acting lncRNAs is as follows: a is ENSACAG00000041900 (locus on chromosome 1), b is ENSACAG00000037173 (locus on chromosome 1), c is ENSACAG00000036367 (locus on chromosome 2), d is ENSACAG00000031293 (locus on chromosome 2), e is ENSACAG00000034833 (locus on chromosome 2), f is ENSACAG00000045045 (locus on chromosome 2), g is ENSACAG00000041129 (locus on chromosome 2), h is ENSACAG00000030666 (locus on chromosome 3), i is ENSACAG00000040072 (locus on chromosome 4), j is ENSACAG00000044525 (locus on chromosome 4), k is ENSACAG00000042080 (locus on chromosome 4), l is ENSACAG00000038902 (locus on chromosome 4), m is ENSACAG00000039554 (locus on chromosome 5), n is ENSACAG00000032218 (locus on chromosome 5), o is ENSACAG00000042324 (locus on chromosome 6), p is ENSACAG00000030570 (locus on chromosome 6), and q is ENSACAG00000044053 (locus on chromosome 6). The lncRNA in the red boxplot (c) is significantly associated with higher coverage of the H4K16ac signal, whereas the lncRNAs in the blue boxplots (f and q) are significantly associated with lower coverage of the H4K16ac signal. Boxplots in dark grey represent H4K16ac coverage from 100,000 random positions. Significant differences, Mann-Whitney U test. Error bars, maximum and minimum values, excluding outliers. *P*-values were corrected using the Benjamin Hochberg correction. N values for the lncRNAs are 18,567, 7525, 2133, 8323, 1176, 14,767, 1489, 2842, 873, 3543, 11,509, 9044, 10,440, 1160, 3159, 1732, 1152. (**f-i**) Same as in (a-d) for female and male brains. (**j**) Expression levels (TPM) from 47 embryonic and 28 adult tissues for ENSACAG0000003636 (in red) associated with a higher signal of H4K16ac and ENSACAG00000044053 and ENSACAG00000045045 (in blue) associated with a lower signal of H4K16ac. (**k**) Expression levels (TPM) from 47 embryonic and 28 adult tissues for the gene targets of ENSACAG0000003636 (in red) and ENSACAG00000044053 and ENSACAG00000045045 (in blue). Significant differences, Mann-Whitney U test. Error bars, maximum and minimum values, excluding outliers. *P*-values were corrected using the Benjamin Hochberg correction. N values are 8112 (c), 15,210 (f), and 35,646 (q)

We identified notable associations by analyzing the trans-acting lncRNAs with the highest number of chromatin contacts. One lncRNA, ENSACAG00000036367, exhibited more frequent interactions with loci enriched in H4K16ac (Fig. 6a-i). Conversely, two other lncRNAs, ENSACAG00000045045 and ENSACAG00000044053, displayed fewer interactions with H4K16ac sites than expected across samples (Fig. 6a-i). We further investigated the transcription profile of these three lncRNAs and found that they are expressed in multiple tissues in both embryos and adults (Fig. 6j).

Additionally, we explored the putative gene targets of these three lncRNAs: ENSACAG00000036367 exhibited contacts with promoter regions of 86 protein-coding genes and 11 lncRNAs, while ENSACAG00000044053 contacted 368 protein-coding genes and 32 lncRNAs. Similarly, ENSACAG00000045045 had interactions with 768 protein-coding genes and 114 lncRNAs. Although functional enrichment analyses did not reveal any overrepresented biological process or metabolic pathway, an intriguing finding emerged. The expression levels of target genes associated with ENSACAG00000036367, which displayed enrichment in H4K16ac, were significantly higher than those of target genes associated with ENSACAG00000044053 and ENSACAG00000045045, which did not exhibit enrichment in H4K16ac (Fig. 6k).

## Discussion

In this study, we explored the ChAR-seq methodology to investigate the contact map of chromatin-interacting RNA molecules in a non-traditional model species, *A. carolinesis*. We encountered several challenges associated with the lack of annotations for many non-coding elements and the need to restrict our analyses to chromosomes 1 to 6 due to incomplete genome assembly. Despite these obstacles, we discovered intriguing patterns regarding the RNAs in *A. carolinesis*, which should inspire future studies into RNA-DNA interactions in other non-traditional model species.

Notably, we observed that certain RNAs, such as ribosomal RNAs, snRNAs, and snoRNAs exhibited multiple interactions across the entire genome. This observation is expected from a successful ChAR-seq experiment and serves as a positive control to validate the reliability of the results. These interactions are likely non-specific, arising from highly transcribed RNA molecules diffusing within the eukaryotic nucleus and being captured in connection with accessible chromatin. It is worth also mentioning that the ChAR-seq method captured significant amounts of nascent transcripts [24], which can also serve as positive controls of the ChAR-seq experiments because they confirm that the method truly trapped RNA molecules that were in contact or in close proximity to adjacent DNA. A recently published technique [32], RADICL-seq, attempts to mitigate the number of nascent transcripts by inhibiting RNA Polymerase II using actinomycin D before cell fixations. Although this technique works effectively with cell cultures, adapting it to bulk tissues from non-model species without available cell lines could pose challenges. Nascent transcripts attached to chromatin may represent regulatory elements within their respective loci. However, differentiating between mature RNAs that regulate their own locus and nascent transcripts attached to chromatin is challenging for *cis*-acting lncRNAs.

Despite the methodological difficulties encountered in working with *A. carolinenesis*, we successfully characterized the type and frequency of contacts made by lncRNAs. The majority of these contacts are either *cis*-proximal or *trans*-acting, exhibiting a pattern distinct from other classes of RNAs, such as ribosomal RNAs, snRNAs, and snoRNAs, which mostly exhibit *trans*-acting interactions. Although lncRNAs may have contacts in *trans* that represent spurious interactions, combining ChAR-seq and ChIP-seq data allowed us to uncover statistical associations that could help differentiate lncRNAs with potential regulatory functions. While our conclusions are based on ChAR-seq data generated from two individuals and may be limited in terms of predicting processes active in a population, the consistency of patterns observed across different types of data (ChAR-seq, ChIP-seq, and RNA-seq) supports the notion that our findings represent general active processes in *A. carolinensis*. Three lncRNAs are of particular interest due to their significant enrichment in chromatin contacts with the H4K16ac epigenetic mark. ENSACAG00000036367 may be involved in gene activation, while ENSACAG00000045045 and ENSACAG00000044053 could play a role in gene silencing. Further work using gene-specific techniques could reveal associated proteins, such as acetyltransferases or methyltransferases complexes.

The results presented in this study provide a partial glimpse into the interaction map between RNA-DNA molecules in *A. carolinensis*. Our data is limited to lncRNAs with broad expression patterns or specifically expressed in the liver. Currently, the liver is the most suitable tissue for ChAR-seq in lizards due to the considerable amount of starting material required and the small size of the organs. Ideally, future studies will integrate ChAR-seq, ChIP-seq, and RNA-seq analyses using the same sample. To further elucidate the functional characterization of annotated lncRNAs, additional data encompassing various tissues and developmental stages, along with other epigenetic modifications, would be necessary. It is worth noting that many lncRNAs are tissue-specific [35–37], and their characterization would require a broader range of experimental data. Moreover, our focus was solely on lncRNAs interacting with DNA, while numerous lncRNAs may interact with other molecules within the cell. Therefore, investigating lncRNAs associated with the proteome would necessitate an all-to-all protein-RNA protocol.

## Methods

### Samples

Two adult *A. carolinensis* individuals, one male and one female, were captured in Tampico, Tamaulipas, Mexico (170 m.a.s.l.; SEMARNAT Scientific Collector Permit 08–043). The animals were housed under controlled conditions, with an ambient temperature of 22 ± 2 °C, relative humidity of 55 ± 15%, and a day/night cycle of 12 h/12 h with live food and water *ad libitum*. The animals were housed together in a large terrarium (50.8 cm width, 40.64 cm depth, 20.32 cm height) equipped with UV light. Prior to the experimental procedure, animals were euthanized using a guillotine. The procedure was performed by an experienced technician. All animal procedures were conducted in accordance with the ethical guidelines of the Bioethical Committee of the Universidad Nacional Autónoma de México. The livers were immediately flash-frozen in liquid nitrogen and stored in 1.5 ml tubes at -80 °C until use. Due to the requirements in starting material, the liver was the most suitable option for the study because it is the largest organ in lizards. The inclusion/exclusion criteria, randomization, blinding/masking, and outcome measures do not apply to this study since the experiment was carried out with two individuals as biological replicates.

### Generation of ChAR-seq data

ChAR-seq is a recent capture method that traps RNA/chromatin interactions [29, 30]. We rapidly homogenized the frozen livers with a mortar and pestle before performing the protein cross-link with formaldehyde (16%, 10 minutes at room temperature, Thermo Scientific, Cat. No. 28908). We then ligated a specific biotinylated linker to the 3’ ends of the RNA molecules using an RNA ligase (T4 RNA Ligase 2, truncated KQ, NEB, Cat. No. M0373L). The top strand of the linker is a 5′-adenylated ssDNA, HPLC purified, ordered at IDT (https://www.idtdna.com/site/home/) as follows: /5rApp/AANNNAAACCGGCGTCCAA GGATCTTTAATTAAGTCGCAG/3SpC3/. The bottom strand is a biotinylated ssDNA, HPLC purified, ordered at IDT as follows: /5Phos/GATCTGCGACTTAATTA AAGATCCTTGGACGCCGG/iBiodT/T). RNA molecules were reverse transcribed (Bst 3.0 DNA Polymerase, NEB, Cat. No. M0374L), the genomic DNA was cut with a restriction enzyme (DpnII, NEB, Cat. No. R0543L), and the 5’ of the adjacent genomic DNA was ligated to the other end of the linker using a DNA ligase (T4 DNA Ligase, HC, Thermo Scientific, Cat. No. EL0013). Proteins were removed using Proteinase K (Thermo Scientific, Cat. No. EO049) and the 2nd strand of the cDNA-linker-DNA molecules was synthesized with Escherichia coli DNA polymerase I (NEB, Cat. No. M0209L). cDNA-linker-DNA molecules were purified with magnetic beads coated with streptavidin (Dynabeads MyOne Streptavidin T1, ThermoFisher, Cat. No. 65601) and prepared for sequencing. The biotinylated linker, which is the key to a successful ChAR-seq experiment, has a 5’ end that contains an adenylated single-stranded sequence that can bind RNA and a 3’ end that contains a recognition site for adjacent DNA fragments that have been digested by DpnII. The linker’s short sequence serves as a molecular tool to control the ligation of RNA and DNA molecules that are in direct contact or in close proximity. More details about the experimental protocol can be found in [29].

### Analysis of ChAR-seq data

One male and one female Illumina TruSeq stranded RNA library were sequenced using an Illumina NovaSeq 6000 machine in Novogene, California. We sequenced 1,020,074,230 (male library) and 9,96,050,284 (female library) 150 nucleotides long paired-end reads. Reads were trimmed using the list of adaptors used in the experimental protocol with trimmomatic (v0.36; parameters: ILLUMINACLIP:illuminaClipping_main.fa:2:30:10 LEADING:3 TRAILING:3 SLIDINGWINDOW:4:15) [38]. Valid reads were obtained by extracting sequences that contained the tag sequence (ACCGGCGTCCAAG) present in the linker or its reverse complement (CTTGGACGCCGGT). The orientation of the tag sequence allowed the identification of the RNA and DNA fragments; the 5’ end before the tag sequence corresponded to the RNA whereas the 3’ end after the tag sequence corresponded to the DNA. Since we sequenced paired-end reads, the orientation of the paired read with the tag sequence relative to the paired read without the tag sequence indicates if the latter represented an RNA or DNA molecule, and was mapped accordingly. DNA fragments longer than 17 base pairs were mapped onto the *A. carolinensis* reference genome (release 104; https://www.ensembl.org) using Bowtie2 (v2.3.4.1; parameters: -p 6 -a -D 20 -R 3 -N 1 -L 18 -i S,1,0.50 --no-unal --no-head --no-sq) [39]. RNA fragments longer than 17 base pairs were mapped to the *A. carolinensis* transcriptome (release 104; https://www.ensembl.org) using Bowtie2 (v2.3.4.1; parameters: -p 6 -a -D 20 -R 3 -N 1 -L 18 -i S,1,0.50 --no-unal --no-head --no-sq) [39]. Since the annotated transcriptome from the Ensembl database could be incomplete, we also mapped using Bowtie2 the RNA fragments to a *de novo* male/female transcriptome generated using Trinity (v2.8.5, parameters: --seqType fq –single –SS_lib_type F –CPU 15 –max_memory 150G) [40]. Finally, RNA fragments longer than 50 base pairs were also mapped to the reference genome using HISAT2 (v2.1.0; parameters: -q --threads 16 -a -N 1 -L 18 -i S,1,0.50 -D 20 -R 3 --pen-noncansplice 15 --mp 1,0) [41]. RNA or DNA fragments that were less than 17 base pairs were not utilized and the paired reads were discarded. We also discarded reads where the RNA or DNA fragments showed two or more top alignments with the same score (multimappers). We verified the redundancy of the genomic coordinates of fragments that mapped to the transcriptome from Ensembl, the *de novo* transcriptome, and the genome using HISAT2. When a fragment was mapped to the same location in the different databases, we chose the coordinates from the Ensembl transcriptome. IDs from the paired-end reads were used to match the RNA and DNA mapping positions. Valid RNA-DNA contacts were defined as fragments that mapped a single time to their respective databases. We obtained 15,520,949 and 45,816,652 valid contacts for the two replicates. We assigned the contacts to specific genes using the annotations from *A. carolinensis* genome (release 104; https://www.ensembl.org). We reorganized the contacts based on the different classes of RNA molecules. We plotted the number of contacts against the type of RNA molecules and using dot plots we plotted the specific positions of the RNA contacts mapped to the transcriptome against the positions of the DNA contacts mapped to the genome using R [42]. For plotting purposes, we concatenated the chromosomes and unassembled scaffolds. We assigned continuous positions starting with chromosomes 1 to 6, then the linkage groups alphabetically, and finally the unassembled scaffolds alphabetically. We also calculated a Contact Distribution Index for each RNA class based on the chromosome with the maximum number of contacts divided by the total number of contacts in all chromosomes. We plotted the values of the index against the type of RNA molecules using R [42]. *Cis*-acting lncRNAs were defined as those having > 50% of their contacts on the same chromosome from which they are transcribed. *Trans*-acting lncRNAs were defined as those having > 50% of their interactions in other chromosomes. We calculated the distance from the locus for the *cis*-acting lncRNA as the absolute difference between the middle point of the genomic position of a lncRNA and all the genomic positions of the start of the contacts on the same chromosome. We also estimated the difference between the middle point of the genomic position of a lncRNA and the genomic positions of its contacts restricted to 20 Kb, 40 Kb, 100 Kb, 200 Kb, and 300 Kb around the lncRNA locus. For *trans*-acting lncRNAs, we limited the analysis to those annotated on the assembled chromosomes 1 to 6 (other scaffolds are too small and tend to have an overestimated number of contacts in *trans*). We mapped the frequency of contacts along the genome, using the positions of the concatenated genome.

### Analysis of ChIP-seq data

We downloaded the reference genome and transcriptome of *A. carolinensis* from the Ensembl database (release 104; https://www.ensembl.org). The results regarding the validity and robustness of the ChIP-seq data were published previously in [34]. We downloaded ChIP data for H4K16ac data for brain and liver of two female replicates and two male replicates from the NCBI-SRA database (PRJNA381064). Liver was also the tissue used for ChAR-seq. ChIP-seq data were trimmed for adaptors and low-quality positions using trim_galore (v0.6.2) (https://github.com/FelixKrueger/TrimGalore). ChIP-seq data were mapped to the *A. carolinensis* reference genome using Bowtie2 (v2.3.4.1) [39]. BAM files were indexed and we estimated RPKM values of their coverage along the entire genome for windows of 50 bp using deepTools2 (v3.3.1) (bamCoverage tool) [43]. We then obtained the RPKM values for the positions where a particular lncRNA was in contact with chromatin. We plotted the RPKM values for all contact sites for the top 17 *trans*-acting lncRNAs using R [42]. Significant differences were estimated using the Mann-Whitney U test. *P*-values were corrected using the Benjamin Hochberg correction. Statistical analyzes were conducted in R [42].

### Analysis of RNA-seq data

We downloaded RNA-seq data for 15 embryonic and 14 adult tissues from females (including liver samples) and 32 embryonic and 14 adult tissues from males (including liver samples) from the NCBI-SRA database (https://www.ncbi.nlm.nih.gov/sra; PRJNA381064). The results regarding the validity and robustness of the RNA-seq data were published previously in [34]. RNA-seq data were trimmed for adaptors and low-quality positions using trim_galore (v0.6.2) (https://github.com/FelixKrueger/TrimGalore). RNA-seq data were aligned to the reference transcriptome using Kallisto [44] to estimate gene expression levels (TPM). We obtained the TPM values for specific lncRNAs and plotted the TPMs from the 15 embryonic and 14 adult tissues using R [42]. We verified that chromatin contacts were at TSS (+- 5Kb). We identified these genes and carried out functional enrichment analyses using the webgestalt platform (http://www.webgestalt.org/). We used chicken as reference species, and we performed over-representation analyses of geneontology, focusing on biological processes (no-redundant) and pathways (KEGG [45]). We used the Ensembl IDs as input data and the genome protein-coding as a reference set. We also used the string-db platform (https://string-db.org/), standard settings, to explore potential interactions among selected genes. We obtained the TPM values of these target genes and plotted their TPMs from the 15 embryonic and 14 adult tissues using R [42]. Significant differences were estimated using the Mann-Whitney U test. Statistical analyzes were conducted in R [42].

### Electronic supplementary material

Below is the link to the electronic supplementary material.

Supplementary Material 1

## Data Availability

All data are available in the main text or the supplementary materials. Sequencing data have been deposited in the NCBI-SRA under BioProject PRJNA880637.
